# Supplementary material for: The Construction and Exploration of a Comprehensive MicroRNA Centered Regulatory Network in Foxtail Millet (Setaria italica L.)
Source: Front Plant Sci. 2022 May 6;13:848474. doi: 10.3389/fpls.2022.848474 (PMC9121102; doi:10.3389/fpls.2022.848474)
Supplement: Supplementary file 7 [file Data_Sheet_7.zip › Sit-miR528_Seita.5G152600.1_2562_TPlot.pdf]

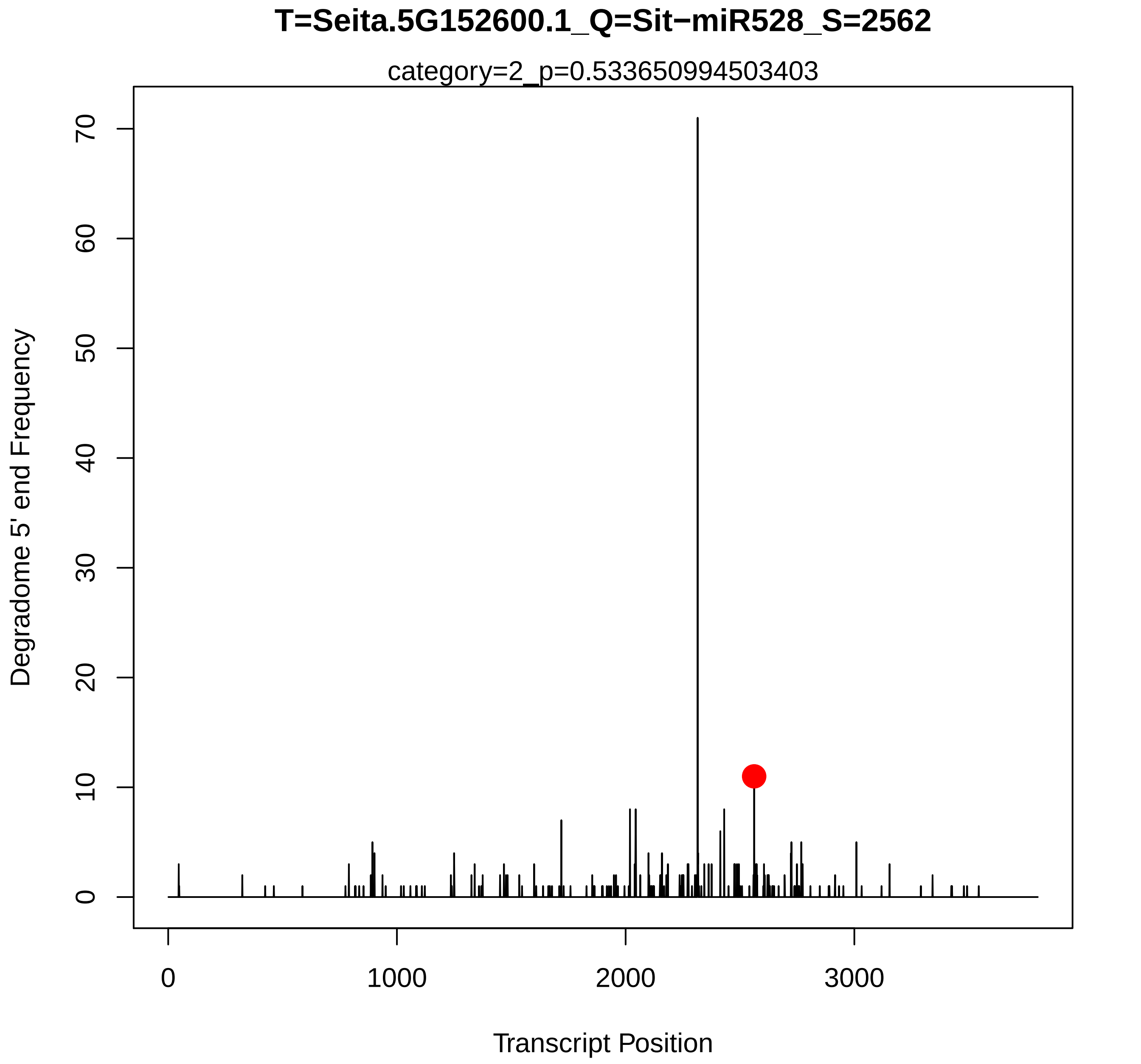
T=Seita.5G152600.1_Q=Sit−miR528_S=2562
category=2_p=0.533650994503403
Degradome 5' end Frequency
0
10
20
30
40
50
60
70
Transcript Position
0
1000
2000
3000
